# Increased Anxiety of Public Situations During the COVID-19 Pandemic: Evidence From a Community and a Patient Sample

**DOI:** 10.32872/cpe.4221

**Published:** 2021-06-18

**Authors:** Andre Pittig, Valentina M. Glück, Juliane M. Boschet, Alex H. K. Wong, Paula Engelke

**Affiliations:** a Department of Psychology (Biological Psychology, Clinical Psychology, and Psychotherapy), University of Würzburg, Würzburg, Germany; b Center of Mental Health, University of Würzburg, Würzburg, Germany; Philipps-University of Marburg, Marburg, Germany

**Keywords:** anxiety, COVID-19, emotional distress, public situations

## Abstract

**Background:**

Increases in emotional distress in response to the global outbreak of the SARS-CoV-2 (COVID-19) pandemic have been reported. So far, little is known about how anxiety responses in specific everyday public life situations have been affected.

**Method:**

Self-reported anxiety in selected public situations, which are relevant in the COVID-19 pandemic, was investigated in non-representative samples from the community (n = 352) and patients undergoing psychotherapy (n = 228). Situational anxiety in each situation was rated on a 5-point Likert scale (0 = no anxiety at all to 4 = very strong anxiety). Situational anxiety during the pandemic was compared with retrospectively reported situational anxiety before the pandemic (direct change) and with anxiety levels in a matched sample assessed before the pandemic (n = 100; indirect change).

**Results:**

In the community and patient sample, indirect and direct change analyses demonstrated an increase in anxiety in relevant public situations but not in control situations. Average anxiety levels during the pandemic were moderate, but 5-28% of participants reported high to very high levels of anxiety in specific situations. Interestingly, the direct increase in anxiety levels was higher in the community sample: patients reported higher anxiety levels than the community sample before, but not during the pandemic. Finally, a higher increase in situational anxiety was associated with a higher perceived danger of COVID-19, a higher perceived likelihood of contracting COVID-19, and stronger symptoms of general anxiety and stress.

**Conclusions:**

Preliminary findings demonstrate an increase in anxiety in public situations during the COVID-19 pandemic in a community and a patient sample. Moderate anxiety may facilitate compliance with public safety measures. However, high anxiety levels may result in persistent impairments and should be monitored during the pandemic.

## Highlights

- Anxiety in public situations has increased in Germany in response to the COVID-19 pandemic.
- Average anxiety levels were moderate, but 5-28% of participants reported high to very high levels of anxiety.
- A stronger increase of anxiety was linked to a higher perceived likelihood and dangerousness of a COVID-19 infection.
- Large-scale representative studies monitoring the development of persistent anxiety are needed.

Emotional distress has increased in response to the global outbreak of the SARS-CoV-2 (COVID-19) pandemic. Moderate to severe increases in distress have been reported internationally, for example, in China, the USA, Canada, Iran, and Europe (e.g., Asmundson et al., 2020; Mazza et al., 2020; Moghanibashi-Mansourieh, 2020; Pierce et al., 2020; Salari et al., 2020; Torales et al., 2020; Wang et al., 2020). While early reports focused on the general increase in emotional distress, more recent studies specifically reported increases in symptoms of anxiety, depression, and stress (Asmundson et al., 2020; Taylor et al., 2020; Torales et al., 2020). To date, little is known about emotional responses in specific public situations that are characterized by an increased threat of COVID-19 infection. These specific emotional responses are, however, important to fully understand emotional responses to the COVID-19 pandemic and how they may influence our daily life.

Public policy measures (i.e., behavioral recommendations or restrictions) to reduce the spread of COVID-19 vary internationally. In Germany, public life was largely “shut down” for approximately four weeks at the beginning of the COVID-19 pandemic (i.e., from mid-March 2020 to mid-April 2020). After COVID-19 infection numbers declined, some restrictions were revoked, but others were continued as the pandemic was ongoing (for German policy measures, see Steinmetz et al., 2020). Especially physical distancing, the use of disinfectant, and wearing face masks were recommended in most public situations (see Robert Koch Institute, 2020). Relevant public situations for COVID-19 related restrictions concerned public transport, restaurants and supermarkets, and effectively every crowded public area. As had been communicated to the general public, these public situations are especially salient for COVID-19 related threats. The resulting threat salience may be linked to elevated situational anxiety in these public situations. In the ongoing pandemic, moderate situational anxiety levels may indeed be adaptive as they may support safety behaviors to prevent COVID-19-related harm (e.g., Arnaudova et al., 2017; Pittig et al., 2020). However, high anxiety levels may also lead to severe distress without additionally supporting safety behaviors and may even persist in the absence of threat (Pittig et al., 2020). Preliminary evidence showed that patients with anxiety-related and mood disorders exhibited stronger COVID-related stress responses than a healthy sample (Asmundson et al., 2020), suggesting that individuals with mental health conditions are prone to experiencing COVID-related anxiety. It is therefore important to explore the potential increase of situational anxiety in public situations during the COVID-19 pandemic, in both general community and clinical samples.

Methodologically, an increase in situational anxiety can be assessed by direct and indirect change measures (Stieglitz & Baumann, 2001). As a measure of direct change, current anxiety levels, which are assessed during the pandemic, can be compared with retrospectively assessed anxiety levels before the pandemic. Retrospective self-reports pose a risk of recall biases (Van den Bergh & Walentynowicz, 2016), whereby recall inaccuracies of affective states might differ between clinical and general community samples (Ben-Zeev, Young, & Madsen, 2009). Nevertheless, this direct approach reflects perceived individual increases in anxiety, i.e., whether individuals *feel* that their anxiety has increased in response to the pandemic. As an indirect change measure, current anxiety levels, which are assessed during the pandemic, can be compared with anxiety levels assessed before the pandemic, optimally within the same sample. The indirect approach is unbiased by retrospective recall but requires repeated measurements. The fast onset of the COVID-19 pandemic prohibited the arrangement of such controlled longitudinal designs. Alternatively, indirect change can be measured by comparing anxiety levels in a sample surveyed during the pandemic with anxiety levels in a different sample assessed before the pandemic. Potential biases caused by differences in certain characteristics between the two samples (e.g., differences in age or biological sex distribution) can be prevented by matching the samples based on these characteristics.

The current study examined both direct and indirect changes in situational anxiety in public situations, which are relevant to the COVID-19 pandemic, in a non-representative community sample and a patient sample. In an online survey, individuals reported their anxiety levels for ten relevant public situations (e.g., taking the bus, going to the supermarket, or being at a crowded public place) and three control situations (e.g., being outdoors alone). We assessed retrospective anxiety levels (i.e., before the pandemic) and current anxiety levels in the previous two weeks (i.e., during the pandemic). Besides comparing these ratings (direct change), situational anxiety during the pandemic was compared with a matched sample that was surveyed before the pandemic (indirect change). To highlight the clinical relevance (i.e., high levels of anxiety may result in impairments), we complemented these analyses by calculating the proportion of individuals who reported high or very high anxiety levels in these situations. We hypothesized that both the community and the patient sample show an increase in situational anxiety during the COVID-19 pandemic, with a stronger increase in the patient sample (Asmundson et al., 2020). Furthermore, we explored the association between increased situational anxiety and symptoms of anxiety, depression, stress, the perceived likelihood of contracting COVID-19, and the perceived dangerousness of a COVID-19 infection. We expected that these clinical symptoms and perceived threat of COVID-19 are positively associated with situational anxiety.

## Method and Materials

### Participants and Recruitment

The study was approved by the local ethics committee (GZEK 2020-31). Three samples of participants anonymously completed an online survey. Participants had to be ≥ 18 years of age. The pre-COVID sample was recruited from the general community before the pandemic (February to April 2019) as part of the validation of an online survey (*n* = 100, Age: *M* = 27.73, *SD* = 10.47, Females: 69.8%). The community sample (*n* = 352, Age: *M* = 35.90, *SD* = 14.09, Females: 69.9%) and the patient sample (*n* = 228, Age: *M* = 39.07, *SD* = 14.50, Females: 60.5%) were recruited during the COVID-19 pandemic (mid of May to mid of July 2020). As present restrictions may influence situational anxiety, we briefly report restrictions that were continuously active across the recruitment period (Steinmetz et al., 2020): Most public situations, e.g., going to supermarkets and shops, using public transport as well as attending religious meetings and demonstrations, were accessible on the condition that specific regulations were followed (e.g., physical distancing, face masks, a limited number of people). Restaurants and entertainment venues (e.g., theaters and cinemas) re-opened stepwise starting between mid of May and mid of June (regionally depending). Meetings of persons from more than two different households were permitted in Germany as from mid of June, but group size was mostly still limited, e.g., to a maximum of ten people. Major public events remained prohibited during the whole recruitment period.

Both the pre-COVID and the community sample were recruited from the general community in Germany via identical online recruitment pathways (e.g., via a German internet platform for online surveys, German local social media groups, and the participant management tool of the University of Würzburg). The patient sample was recruited via the outpatient clinic for psychotherapy at the University of Würzburg. 109 out of 689 participants completed opt-in informed consent but discontinued the survey before providing anxiety ratings for at least one situation and were thus excluded (15.8%). The remaining 580 participants in the community and patient sample completed all situational anxiety ratings, i.e., there were no missing data for the variables of interest, as the completion of sociodemographic data, trait anxiety, and symptom measures was required before answering the situational anxiety ratings. All patients had provided written informed consent to be contacted for research purposes prior to the study and were currently undergoing psychotherapeutic treatment. A total of 496 patients was invited to participate in the study (response rate = 46.0%). The distribution of main primary diagnoses within the invited patients was 33.4% affective disorders, 23.7% anxiety disorders, 15.3% adjustment disorder, 7.4% somatoform disorders, 5.0% obsessive-compulsive disorder, 3.9% posttraumatic stress disorder, 2.9% eating disorders.

### Online Survey

The online survey measured self-reported anxiety in selected public situations, trait anxiety, symptoms of emotional distress, and basic demographic data (i.e., age, sex, employment status). Trait anxiety was assessed with the anxiety subscale of the NEO-PI-R (N1 subscale; Costa & McCrae, 1992). Symptoms of anxiety, depression, and stress over the previous week were assessed with the German short version of the Depression Anxiety Stress Scales (DASS-21; Lovibond & Lovibond, 1995; Nilges & Essau, 2015). All participants, including the pre-COVID sample, completed these two questionnaires. The community and patient sample additionally rated the perceived dangerousness of COVID-19 (5-point Likert-scale from *very harmless* to *very dangerous*) and the subjective likelihood of contracting COVID-19 (5-point Likert-scale from *very unlikely* to *very likely*).

Self-reported anxiety was assessed for 13 selected public situations, mostly taken from a well-established questionnaire for agoraphobia (Mobility Inventory; Chambless et al., 1985). Ten of these situations were regarded as highly relevant in the COVID-19 pandemic: taking the bus, taking the train, going to the supermarket, going to the cinema/theater, shopping mall, restaurant, waiting in line, talking to others, and being at an outdoor or indoor public area with people. Three additional situations were used to control whether general changes in anxiety occurred in situations that are unrelated to COVID-19 but may still provoke some anxiety, i.e., being alone in an unknown area. All participants were instructed to rate their anxiety level for each situation during the previous two weeks (5-point Likert scale; 0 = *no anxiety at all* to 4 = *very strong anxiety*). The community and patient samples retrospectively rated each situation regarding how anxious they were before the COVID-19 outbreak. If participants had not approached a particular situation in the previous two weeks, they were asked to imagine being in the situation and rate the anxiety level accordingly.

### Statistical Analysis

The main research aim was to examine changes in self-reported anxiety in public situations during the COVID-19 pandemic. To this end, we calculated the direct and indirect change in self-reported anxiety. Direct change was analyzed by comparing anxiety ratings for the 13 selected public situations during the previous two weeks with retrospectively reported anxiety for these situations before the pandemic (within-subjects comparison). Therefore, we conducted repeated measures ANOVAs for each situation with Group (community vs. patient sample) as between-subjects factor and Time (previous two weeks vs. before COVID-19) as within-subjects factor, including all participants from both samples recruited during the COVID-19 pandemic. Indirect change was analyzed by comparing anxiety ratings in the previous two weeks in the community and patient sample separately with anxiety ratings for the same situations in the matched pre-COVID sample (between-subjects comparison). As these indirect change analyses may be biased due to different sample characteristics, we aimed to reduce sample bias by matching participants. Precisely, we matched the three samples on age, sex, and employment status using nearest neighbor matching (Ho et al., 2011). As the smallest sample (i.e., the pre-COVID sample) included 100 participants, we selected the closest neighbors in the other samples, respectively. As a result, the indirect change analyses were conducted with 100 participants per sample. Analyses with the complete, but unmatched samples yielded the same pattern of results. Indirect change was analyzed using a MANOVA with anxiety ratings in the previous two weeks in the 13 situations as dependent variables, followed by one-way ANOVAs for each situation with the between-subjects factor Group (pre-COVID, community, patient). Bonferroni-Holm correction was applied in all analyses. Cohen’s *d* and eta-squared are reported as effect sizes.

To highlight the clinical relevance of these analyses, we aimed to provide descriptive data on the frequency of high anxiety levels in public situations in response to the COVID-19 pandemic. For each situation, we calculated the relative number of participants from the complete sample who indicated “strong” or “very strong” anxiety. Finally, we exploratorily examined the associations between the increase in self-reported anxiety (difference score: anxiety during COVID-19 – anxiety before COVID-19) and clinical variables (trait anxiety, symptoms of depression, stress, and anxiety) as well as COVID-19 related variables (perceived dangerousness and likelihood of contracting COVID-19) in the unmatched community and patient samples. To this end, robust winsorized correlations (trim = 0.2) were calculated using the WRS2 package (Mair & Wilcox, 2020) in R (R Core Team, 2020).

## Results

### Increased Anxiety of Public Situations

#### Direct Change

**Figure 1:**
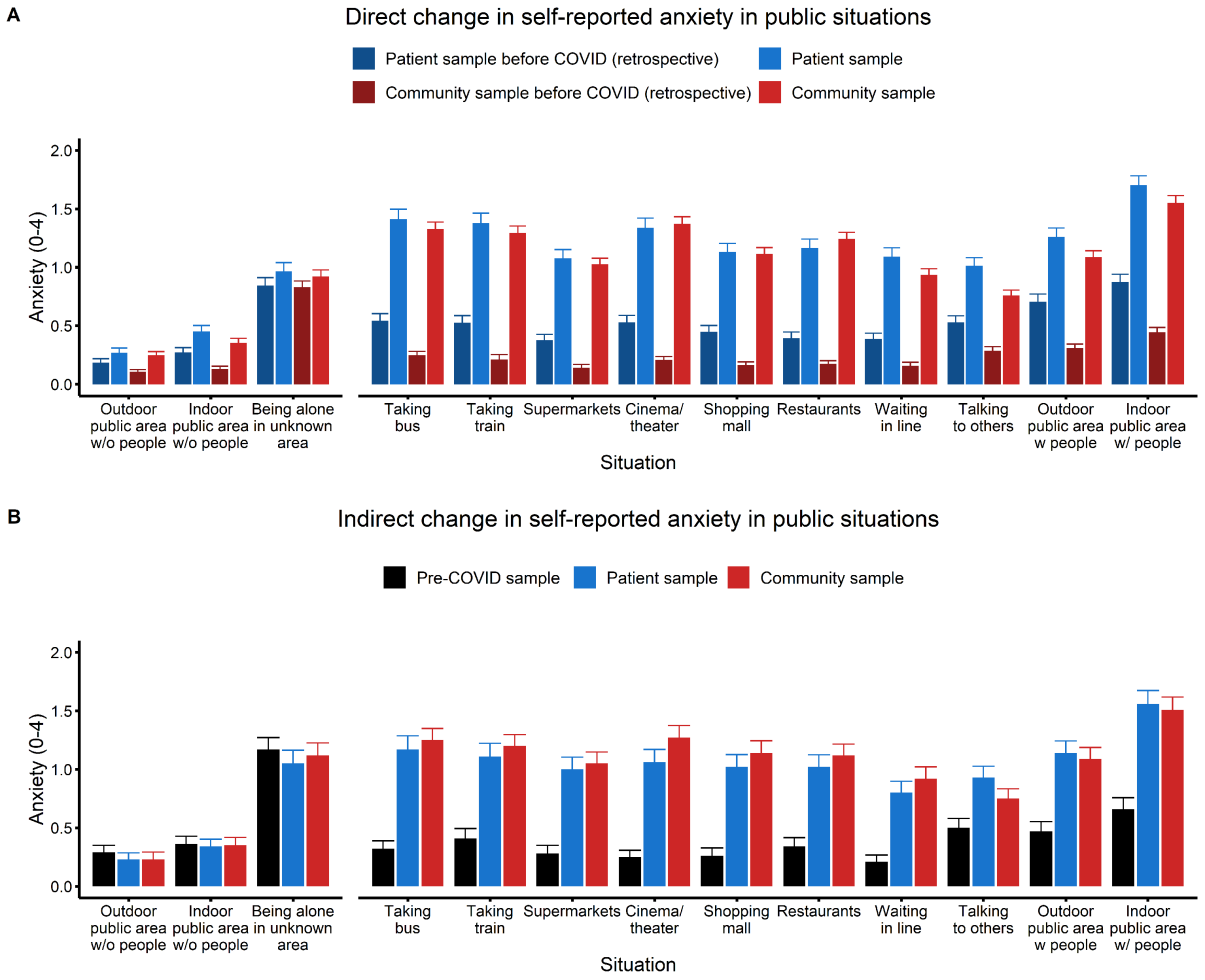
Average Self-Reported Anxiety in Selected Public Situations Before and During the COVID-19 Pandemic (With Standard Error of the Mean) *Note.* Situational anxiety was rated for each situation on a 5-point Likert scale (0 = no anxiety at all to 4 = very strong anxiety). A: Direct change as indicated by comparing anxiety ratings during the previous two weeks (during the pandemic) with retrospectively reported anxiety before the pandemic (within-subject comparison; community sample: *n* = 352, patient sample: *n* = 228). B: Indirect change as analyzed by comparing anxiety ratings for the previous two weeks in a matched community and patient sample with anxiety ratings in the matched pre-COVID sample (between-subject comparison, *n* = 100 for each subsample).

For all situations, there was an increase in self-reported anxiety during the COVID-19 pandemic (see Figure 1A and Table 1). For the control situations, this increase was relatively small and there were no significant effects involving Group. For most COVID-relevant situations, repeated measures ANOVAs yielded a significant interaction of Group and Time. Post-hoc Wilcoxon tests indicated that anxiety increased in all situations in the patient sample, *p*s < .001, *r*s = .86 to 1.00, and in the community sample, *p*s < .001, *r*s = .81 to 1.00. The patient compared to the community sample reported higher retrospective anxiety before the COVID-19 pandemic in most situations, *U*s > 42606.0, *p*s < .020, *r*s = .06 to .25, except for “being alone in an unknown area”, *U* = 39955.0, *p* = .924, *r* = .04. Interestingly, the groups did not differ in anxiety during the COVID-19 pandemic, *U*s < 42858.0, *p*s > .077, *r*s = -.05 to .07.

**Table 1:** Overview of Statistical Results for Direct and Indirect Change

Direct change				Indirect change			
Situation / Effect	*F*	*p*	η^2^	Effect	*F*	*p*	η^2^
Outdoor public area w/o people							
Time	41.32	< .001	.011	Group	0.34	.710	.002
Group	1.43	.232	.002				
Time*Group	2.80	.095	< .001				
Indoor public area w/o people							
Time	72.25	< .001	.024	Group	0.02	.997	< .001
Group	6.12	.014	.008				
Time*Group	0.88	.349	< .001				
Being alone in unknown area							
Time	37.33	< .001	.003	Group	0.32	.729	.002
Group	0.10	.755	< .001				
Time*Group	0.67	.413	< .001				
Taking bus							
Time	408.01	< .001	.188	Group	28.32	< .001	.160
Group	7.80	.005	.007				
Time*Group	4.79	.029	.002				
Taking train							
Time	342.30	< .001	.174	Group	19.30	< .001	.115
Group	8.17	.004	.007				
Time*Group	4.80	.029	.002				
Supermarkets							
Time	352.66	< .001	.173	Group	22.33	< .001	.131
Group	6.20	.013	.006				
Time*Group	4.64	.032	.002				
Cinema/theater							
Time	390.67	< .001	.194	Group	32.62	< .001	.180
Group	4.71	.030	.004				
Time*Group	12.86	< .001	.006				
Shopping mall							
Time	357.68	< .001	.170	Group	25.31	< .001	.146
Group	6.10	.014	.006				
Time*Group	9.39	.002	.004				
Restaurant							
Time	364.73	< .001	.197	Group	20.82	< .001	.123
Group	1.49	.223	.001				
Time*Group	9.45	.002	.005				
Waiting in line							
Time	311.01	< .001	.149	Group	18.66	< .001	.112
Group	10.23	.001	.010				
Time*Group	0.68	.409	< .001				
Talking to others							
Time	222.51	< .001	.071	Group	6.12	.002	.040
Group	15.43	< .001	.019				
Time*Group	0.03	.865	< .001				
Outdoor public area w/o people							
Time	283.91	< .001	.106	Group	15.37	< .001	.094
Group	16.99	< .001	.019				
Time*Group	8.17	.004	.003				
Indoor public area w/o people							
Time	398.88	< .001	.167	Group	22.48	< .001	.131
Group	15.08	< .001	.015				
Time*Group	8.28	.004	.003				

*Note.* The factor *Time* refers to the within-subject factor for ratings before (retrospective) vs. during pandemic. The factor *Group* refers to community vs. patient sample (direct change) or pre-COVID vs. community vs. patient sample (indirect change).

This overall pattern differed only for the situations “waiting in line” and “talking to others”. For both, anxiety was higher during than before the pandemic (Table 1), and the patient sample reported higher anxiety. However, there was no significant interaction between Group and Time.

In sum, direct change analyses indicated a slight increase in self-reported anxiety in the control situations and a larger increase in all COVID-relevant public situations. Interestingly, the latter increase was higher in the community sample compared with the patient sample, as indicated by patients’ higher anxiety levels before but not during the pandemic in most public situations.

#### Indirect Change

For the matched samples, the significant MANOVA, Pillais’ Trace = .33, *F*(26, 572) = 4.27, *p* < .001, was followed up by one-way ANOVAs for each situation, comparing self-reported anxiety levels during the previous two weeks between the three samples. As expected, no significant differences were found for the three control situations (see Figure 1B and Table 1). In all COVID-relevant public situations, self-reported anxiety during the previous two weeks differed between groups. For almost all situations, anxiety ratings did not differ between the community and the patient sample, *t*s < 1.58, *p*s > .116, *d*s = -0.19 to 0.05, but were higher than in the pre-COVID sample, respectively, *t*s > 4.61, *p*s < .001, *d*s = 0.68 to 1.20. This pattern only differed for the situation “talking to others”: While the patient sample again reported higher anxiety than the pre-COVID sample, *t* = 3.48, *p* = .002, *d* = 0.48, the community sample did not differ from the other two samples, *ts* < 2.03, *ps* > .087, *ds* < 0.30. In sum, indirect change analyses of the matched samples indicated higher self-reported anxiety levels during the previous two weeks than before the COVID-19 pandemic in all relevant public situations.

### Frequency of High and Very High Anxiety in Public Situations

The proportion of individuals indicating high or very high anxiety levels is displayed in Table 2. Overall, the frequency of high or very high anxiety increased by approximately 10%. In the community sample, the average increase was 8% (indirect) to 10% (direct). In the patient sample, the average increase was 11% (direct) to 12% (indirect).

**Table 2:** Relative Frequency of High or Very High Anxiety to Distinct Public Situations

Public situation	Community sample (*n* = 352)		Patient sample (*n* = 228)		Pre-COVID sample (*n* = 100)
	During^a^	(Before)^b^	During^a^	(Before)^b^	Before^a^
Outdoor public place w/o people	0.9%	(0.3%)	1.8%	(0.9%)	0.0%
Indoor public place w/o people	2.3%	(0.6%)	1.8%	(1.3%)	2.0%
Being alone in unknown area	8.5%	(7.1%)	10.5%	(7.5%)	18.0%
Taking bus	15.1%	(1.4%)	19.3%	(4.8%)	4.0%
Taking train	15.1%	(1.1%)	18.9%	(5.3%)	2.0%
Supermarkets	7.7%	(0.6%)	11.0%	(2.6%)	4.0%
Cinema/theater	15.6%	(1.4%)	20.6%	(6.1%)	0.0%
Shopping mall	10.5%	(0.9%)	11.0%	(3.9%)	4.0%
Restaurants	13.1%	(0.9%)	12.7%	(3.5%)	6.0%
Waiting in line	5.7%	(1.1%)	11.8%	(3.1%)	2.0%
Talking to others	5.1%	(1.1%)	9.2%	(3.9%)	2.0%
Outdoor public area w/ people	8.8%	(1.1%)	15.8%	(6.6%)	8.0%
Indoor public area w/ people	20.5%	(3.1%)	27.6%	(7.9%)	6.0%

*Note.* Proportion of participants responding with “strong anxiety” or “very strong anxiety” in the different public situations.

^a^Anxiety during the previous two weeks.

^b^Retrospective anxiety before the COVID-19 pandemic.

### Associations Between Anxiety Increase, Symptoms, and COVID-19 Related Variables

Robust winsorized correlations within the patient and the community samples are shown in Table 3. Most correlations were similar in both samples. A stronger increase in self-reported anxiety (i.e., a higher direct change score) was associated with a higher perceived dangerousness and a higher perceived likelihood of contracting COVID-19 (the latter two correlated positively in the patient sample, *r* = .41, *p* < .001, and in the community sample, *r* = .33, *p* = .003). Moreover, a stronger increase in self-reported anxiety was associated with stronger symptoms of anxiety and stress, but not with symptoms of depression, or with trait anxiety.

**Table 3:** Associations Between Direct Increase of Anxiety in Public Situations and COVID-19 Variables, Clinical, and Demographic Data

Sample	COVID-19 variable		Clinical variable			
	Danger	Likelihood contraction	Trait Anxiety	Anxiety	Stress	Depression
Community sample	.25*	.19	.16	.21*	.23*	.03
Patient sample	.26*	.26*	.12	.21*	.28*	.14

*Note.* Zero-order robust winsorized correlations (trim = 0.2) with direct change score (anxiety during COVID-19 minus before COVID-19).

*p < .05.

## Discussion

The current study investigated changes in anxiety in public situations in response to the COVID-19 pandemic. In all relevant public situations, anxiety increased strongly, both in a community sample and in a clinical sample of patients affected by mental disorders. In both samples, evidence for increased anxiety was supported by direct and indirect change analyses. For direct change, levels of situational anxiety during the pandemic were higher than retrospective anxiety levels of the same individuals before the pandemic. For indirect change, situational anxiety during the pandemic was higher than anxiety in the same situations assessed before the pandemic in a matched community sample. Thus, the present findings expand previous reports concerning an increase in general emotional distress during the COVID-19 pandemic (e.g., Asmundson et al., 2020; Taylor et al., 2020), as the current results highlight a distinct increase in self-reported anxiety in COVID-relevant public situations.

The increase in situational anxiety in response to the pandemic was not driven by outdoor situations per se. No strong increase in anxiety was found in situations that do not involve potential physical contact with others (e.g., being alone in a public area). In these control situations, self-reported anxiety during the pandemic was only slightly higher than retrospectively reported anxiety. Also, anxiety levels in these control situations before the pandemic and during the pandemic did not differ. Thus, increased situational anxiety was linked to physical closeness to other individuals, presumably due to the associated risk of contracting COVID-19. In support, a higher perceived likelihood of contracting COVID-19 and a higher perceived danger of COVID-19 infections were associated with a stronger increase in situational anxiety. In sum, increased anxiety of public situations likely resulted from a higher perceived threat of contracting COVID-19.

Average situational anxiety levels during the pandemic were moderate. As the ongoing pandemic represents a realistic threat to the individual and the society, moderate levels of anxiety in situations that pose a higher risk of contraction can be seen as adaptive responses. Anxiety activates the defensive network and facilitates defensive behaviors such as avoidance or safety behavior (Pittig et al., 2018, 2020). In this regard, moderate anxiety levels could promote compliance with safety measures. However, extremely high anxiety levels may not entail additional benefits for preventing infections but may lead to severe distress and impairments. On average, there was an increase of 8-12% in individuals who reported high to very high anxiety in public situations. Up to 20-28% of participants indicated high or very high anxiety when being in an indoor public area with others during the pandemic. Importantly, high anxiety levels may result in avoidance of relevant situations, which may persist even in the absence of threat (Pittig et al., 2020). It therefore seems important to identify individuals with high anxiety and to monitor the development of persistent maladaptive anxiety and potential avoidance. Notably, individuals who perceived COVID-19 as being more dangerous and perceived the likelihood of contracting COVID-19 as being higher showed a stronger increase in situational anxiety. Moreover, a stronger increase in situational anxiety has been linked to stronger general symptoms of stress and anxiety. These findings suggest that caution should be placed on these individuals, given that they are more likely to experience a higher level of psychological distress and detrimental effects on their overall well-being (Kang et al., 2020; Torales et al., 2020).

Interestingly, there were some expected, but also unexpected, differences between the community and the patient sample. As expected, patients reported higher levels of retrospective anxiety than participants of the community sample. These heightened anxiety levels before the COVID-19 outbreak may reflect higher perceived threat in these situations due to relevant psychopathologies (e.g., agoraphobia, social anxiety). However, no group differences in situational anxiety during the pandemic were observed. In other words, both samples showed similar anxiety levels in public situations during the COVID-19 pandemic. Importantly, the lack of group differences was not due to a ceiling effect, considering that the average self-reported anxiety was moderate in both samples. These results are not in line with previous findings of higher levels of COVID-19-related distress in clinical samples than in the general population (Asmundson et al., 2020). There may be multiple explanations. First, whereas previous studies assessed general emotional distress, the present study examined anxiety in specific public situations. The higher levels of general distress found in previous studies may be caused by factors different from anxious responding in COVID-relevant situations (e.g., troubles coping with self-isolation, general worries about the future, or the socio-economic impact of COVID-19; see Asmundson et al., 2020). Second, the patient sample consisted of patients with mental disorders undergoing cognitive-behavioral treatment. The ongoing treatment may have buffered negative effects of the pandemic and facilitated adaptive coping strategies. Third, patients and non-patients may have applied diverging scaling in COVID-related anxiety ratings (e.g., patients who have frequently experienced highly anxious states may classify levels of anxiety as “moderate” when non-patients may classify similar levels as “high”). Finally, the lack of differences between the patient and community sample under realistic threat is in line with findings from experimental fear learning research. Specifically, a meta-analysis found no differences in learning novel fear responses to a stimulus signaling threat between healthy individuals and patients with anxiety disorders (Duits et al., 2015). However, patients showed elevated responses to a safety signal and ongoing fear responses in the absence of threat. Thus, patients seemingly do not show elevated responses to stimuli and situations signaling realistic threat but rather show a bias to stimuli and situations signaling safety or the absence of previous threat. Therefore, it is important to monitor increased anxiety responses in patients when the risk for contraction of COVID-19 decreases. Moreover, the present study did neither assess the effects of psychotherapy on the negative psychological effects of the COVID-19 pandemic, nor did it assess potential increases in anxiety in currently untreated clinical samples. Thus, additional research is warranted.

The present results are limited by the non-representative samples, which were recruited from a German-speaking population. The generalizability to other populations requires further research. The current findings may only represent a subset of the population but provide the insight that at least in this portion of the German population, an increase in COVID-19-related situational anxiety occurred. As no data about the current place of the participants’ residence were collected, the potential influence of regional variances in COVID-19 incidence values and, relatedly, official regulations at the time of the survey on situational anxiety cannot be ruled out. However, incidences were generally low in Germany and did not exceed 25 per 100,000 population in any German state at the period of the survey (Robert Koch Institute, 2021) and official restrictions did not differ substantially between German regions (see Steinmetz et al., 2020). The study’s results may also be used to generate more elaborate hypotheses on the associations between COVID-19-related and clinical variables on the one side and an increase in situational anxiety on the other side. As outlined above, monitoring general and situation-specific anxiety levels and identifying individuals at risk for developing persistent anxiety and impairments is important for understanding and potentially preventing pandemic-related psychological distress. Public policymakers should facilitate appropriate large-scale, long-term studies. Another limitation is the missing assessment whether participants experienced the public situations during the previous two weeks or whether they imagined being in the situations. Future research may disentangle these potentially diverging responses. Finally, the patient sample was diagnosed with heterogeneous mental disorders, which could not be matched to situational anxiety changes. Thus, we could not evaluate whether there were any differences between different mental disorders or whether a specific disorder may be linked to a higher recall bias.

In conclusion, the current study provides preliminary evidence for an increase in situational anxiety in public situations in a community and a patient sample during the COVID-19 pandemic. Both groups showed similar levels of moderate situational anxiety, which may facilitate compliance with public safety recommendations and restrictions for preventing COVID-19 contractions. However, some individuals display high levels of anxiety, which should be monitored during and after the pandemic.

## References

[r1] Arnaudova, I., Kindt, M., Fanselow, M., & Beckers, T. (2017). Pathways towards the proliferation of avoidance in anxiety and implications for treatment. Behaviour Research and Therapy, 96, 3–13. 10.1016/j.brat.2017.04.004 28457483

[r2] Asmundson, G. J. G., Paluszek, M. M., Landry, C. A., Rachor, G. S., McKay, D., & Taylor, S. (2020). Do pre-existing anxiety-related and mood disorders differentially impact COVID-19 stress responses and coping? Journal of Anxiety Disorders, 74, 102271. 10.1016/j.janxdis.2020.102271 32673930 PMC7342169

[r3] Ben-Zeev, D., Young, M. A., & Madsen, J. W. (2009). Retrospective recall of affect in clinically depressed individuals and controls. Cognition and Emotion, 23(5), 1021–1040. 10.1080/02699930802607937

[r4] Chambless, D. L., Caputo, G. C., Jasin, S. E., Gracely, E. J., & Williams, C. (1985). The Mobility Inventory for Agoraphobia. Behaviour Research and Therapy, 23(1), 35–44. 10.1016/0005-7967(85)90140-8 3985915

[r5] Costa, P. T., & McCrae, R. R. (1992). *NEO PI-R Professional Manual: Revised NEO Personality Inventory (NEO PI-R) and NEO Five-Factor Inventory (NEO-FFI)*. Psychological Assessment Resources.

[r6] Duits, P., Cath, D. C., Lissek, S., Hox, J. J., Hamm, A. O., Engelhard, I. M., van den Hout, M. A., & Baas, J. M. P. (2015). Updated meta-analysis of classical fear conditioning in the anxiety disorders. Depression and Anxiety, 32(4), 239–253. 10.1002/da.22353 25703487

[r7] Ho, D. E., Imai, K., King, G., & Stuart, E. A. (2011). MatchIt: Nonparametric preprocessing for parametric causal inference. Journal of Statistical Software, 42(8). 10.18637/jss.v042.i08

[r8] Kang, L., Ma, S., Chen, M., Yang, J., Wang, Y., Li, R., Yao, L., Bai, H., Cai, Z., Xiang Yang, B., Hu, S., Zhang, K., Wang, G., Ma, C., & Liu, Z. (2020). Impact on mental health and perceptions of psychological care among medical and nursing staff in Wuhan during the 2019 novel coronavirus disease outbreak: A cross-sectional study. Brain, Behavior, and Immunity, 87, 11–17. 10.1016/j.bbi.2020.03.028 32240764 PMC7118532

[r9] Lovibond, P. F., & Lovibond, S. H. (1995). The structure of negative emotional states: Comparison of the Depression Anxiety Stress Scales (DASS) with the Beck Depression and Anxiety Inventories. Behaviour Research and Therapy, 33(3), 335–343. 10.1016/0005-7967(94)00075-U 7726811

[r10] Mair, P., & Wilcox, R. (2020). Robust statistical methods in R using the WRS2 package. Behavior Research Methods, 52(2), 464–488. 10.3758/s13428-019-01246-w 31152384

[r11] Mazza, C., Ricci, E., Biondi, S., Colasanti, M., Ferracuti, S., Napoli, C., & Roma, P. (2020). A nationwide survey of psychological distress among Italian people during the COVID-19 pandemic: Immediate psychological responses and associated factors. International Journal of Environmental Research and Public Health, 17(9), 3165. 10.3390/ijerph17093165 32370116 PMC7246819

[r12] Moghanibashi-Mansourieh, A. (2020). Assessing the anxiety level of Iranian general population during COVID-19 outbreak. Asian Journal of Psychiatry, 51, 102076. 10.1016/j.ajp.2020.102076 32334409 PMC7165107

[r13] Nilges, P., & Essau, C. (2015). Die Depressions-Angst-Stress-Skalen. Schmerz, 29(6), 649–657. 10.1007/s00482-015-0019-z 26205682

[r14] Pierce, M., Hope, H., Ford, T., Hatch, S., Hotopf, M., John, A., Kontopantelis, E., Webb, R., Wessely, S., McManus, S., & Abel, K. M. (2020). Mental health before and during the COVID-19 pandemic: A longitudinal probability sample survey of the UK population. The Lancet: Psychiatry, 7(10), 883–892. 10.1016/S2215-0366(20)30308-4 32707037 PMC7373389

[r15] Pittig, A., Treanor, M., LeBeau, R. T., & Craske, M. G. (2018). The role of associative fear and avoidance learning in anxiety disorders: Gaps and directions for future research. Neuroscience and Biobehavioral Reviews, 88(February), 117–140. 10.1016/j.neubiorev.2018.03.015 29550209

[r16] Pittig, A., Wong, A. H. K., Glück, V. M., & Boschet, J. M. (2020). Avoidance and its bi-directional relationship with conditioned fear: Mechanisms, moderators, and clinical implications. Behaviour Research and Therapy, 126, 103550. 10.1016/j.brat.2020.103550 31981801

[r17] R Core Team. (2020). *R: A language and environment for statistical computing.* R Foundation for Statistical Computing. https://www.r-project.org/

[r18] Robert Koch Institute (2020). Mund-Nasen-Bedeckung im öffentlichen Raum als weitere Komponente zur Reduktion der Übertragungen von COVID-19. Epidemiologisches Bulletin, 19, 3–5. 10.25646/6731

[r19] Robert Koch Institute (2021). Gesamtübersicht der pro Tag ans RKI übermittelten Fälle, Todesfälle und 7-Tage-Inzidenzen nach Bundesland und Landkreis (28.1.2021) [Comrehensive overview of the cases, deaths and 7-day incidences transmitted to the RKI per day by federal state and district (1/28/2021). https://www.rki.de/DE/Content/InfAZ/N/Neuartiges_Coronavirus/Daten/Fallzahlen_Kum_Tab.html;jsessionid=96A314A6010A8C27B84B4FA5B1E042DE.internet061

[r20] Salari, N., Hosseinian-Far, A., Jalali, R., Vaisi-Raygani, A., Rasoulpoor, S., Mohammadi, M., Rasoulpoor, S., & Khaledi-Paveh, B. (2020). Prevalence of stress, anxiety, depression among the general population during the COVID-19 pandemic: A systematic review and meta-analysis. Globalization and Health, 16(1), 57. 10.1186/s12992-020-00589-w 32631403 PMC7338126

[r21] Steinmetz, H., Batzdorfer, V., & Bosnjak, M. (2020, June). *The ZPID lockdown measures dataset for Germany*. ZPID (Leibniz Institute for Psychology Information). 10.23668/PSYCHARCHIVES.3019

[r22] Stieglitz, R.-D., & Baumann, U. (2001). Veränderungsmessung. In *Psychodiagnostik in Klinischer Psychologie, Psychiatrie, Psychotherapie* (S. 21–38). Thieme.

[r23] Taylor, S., Landry, C. A., Paluszek, M. M., Fergus, T. A., McKay, D., & Asmundson, G. J. G. (2020). COVID stress syndrome: Concept, structure, and correlates. Depression and Anxiety, 37(8), 706–714. 10.1002/da.23071 32627255 PMC7362150

[r24] Torales, J., O’Higgins, M., Castaldelli-Maia, J. M., & Ventriglio, A. (2020). The outbreak of COVID-19 coronavirus and its impact on global mental health. The International Journal of Social Psychiatry, 66(4), 317–320. 10.1177/0020764020915212 32233719

[r25] Van den Bergh, O., & Walentynowicz, M. (2016). Accuracy and bias in retrospective symptom reporting. Current Opinion in Psychiatry, 29(5), 302–308. 10.1097/YCO.0000000000000267 27427854

[r26] Wang, C., Pan, R., Wan, X., Tan, Y., Xu, L., Ho, C. S., & Ho, R. C. (2020). Immediate psychological responses and associated factors during the initial stage of the 2019 Coronavirus Disease (COVID-19) epidemic among the general population in China. International Journal of Environmental Research and Public Health, 17(5), 1729. 10.3390/ijerph17051729 32155789 PMC7084952

